# The Submaxillary Ganglion

**Published:** 1865-02

**Authors:** 


### *The Submaxillary Ganglion.*

M. Claude Bernard, in a late communication to the Academy of Sciences, treated of the special function of the submaxilary ganglion. The results of his experiments prove that the tongue is connected with the submaxillary gland by two kinds of nervous arcs, which are, in a measure, concentric; the one of the greatest extent passing through the brain, the other, far shorter, passing through the submaxillary ganglion. There results from these two nervous connections two kinds of reflex action destined to affect the submaxillary gland. The first, which traverses the brain, is *intelligent*, and is excited more particularly by the gustatory faculty of the tongue; the second, which is unintelligent, is transmitted by the submaxillary ganglion, and seems to be prov[illegible]d more especially by the dry or moist condition of the bucco-lingual mucous membrane. The submaxillary ganglion not only possesses the faculty of developing reflex action, which may, by its mediation, reach the gland without passing through the encephalic centre, but it also exerts a special influence upon the intermittent character of the salivary secretion. Everyone is aware of the fact, that the flow of the saliva takes place in an intermittent form, and that only when an exciting cause, reflex or direct, calls forth the activity of the gland, and that the secretion ceases when the exciting cause is withdrawn. M. Bernard has observed that after the section of the submaxillary ganglion, the lingual and chorda tympani nerves still remaining intact, the secretion of the submaxillary gland becomes continuous, although it may be augmented by the application of strong-tasted stimulants to the surface of the tongue. Another fact of interest brought to light by the same experiment is, that when separated from the encephalic centre, the ganglion after a certain time loses its controlling influence over the secretive powers of the gland, which instead of ceasing its function, remains in a condition of permanent activity.—From which it results that the submaxillary ganglion is at one and the same time independent of, and subordinate to, the encephalic centre.

